# A stochastic inference of *de novo* CNV detection and association test in multiplex schizophrenia families

**DOI:** 10.3389/fgene.2013.00185

**Published:** 2013-09-23

**Authors:** Shi-Heng Wang, Wei J. Chen, Yu-Chin Tsai, Yung-Hsiang Huang, Hai-Gwo Hwu, Chuhsing K. Hsiao

**Affiliations:** ^1^Institute of Epidemiology and Preventive Medicine, College of Public Health, National Taiwan UniversityTaipei, Taiwan; ^2^Department of Public Health, College of Public Health, National Taiwan UniversityTaipei, Taiwan; ^3^Genetic Epidemiology Core Laboratory, Division of Genomic Medicine, Research Center for Medical Excellence, National Taiwan UniversityTaipei, Taiwan; ^4^Institute of Brain and Mind Sciences, College of Medicine, National Taiwan UniversityTaipei, Taiwan; ^5^National Applied Research Laboratories, National Center for High-Performance ComputingHsinchu, Taiwan; ^6^Department of Psychiatry, College of Medicine and National Taiwan University Hospital, National Taiwan UniversityTaipei, Taiwan; ^7^Bioinformatics and Biostatistics Core, Division of Genomic Medicine, Research Center for Medical Excellence, National Taiwan UniversityTaipei, Taiwan

**Keywords:** Bayesian model, CNV association test, *de novo* CNV detection, schizophrenia multiplex family, random mutation parameter

## Abstract

The copy number variation (CNV) is a type of genetic variation in the genome. It is measured based on signal intensity measures and can be assessed repeatedly to reduce the uncertainty in PCR-based typing. Studies have shown that CNVs may lead to phenotypic variation and modification of disease expression. Various challenges exist, however, in the exploration of CNV-disease association. Here we construct latent variables to infer the discrete CNV values and to estimate the probability of mutations. In addition, we propose to pool rare variants to increase the statistical power and we conduct family studies to mitigate the computational burden in determining the composition of CNVs on each chromosome. To explore in a stochastic sense the association between the collapsing CNV variants and disease status, we utilize a Bayesian hierarchical model incorporating the mutation parameters. This model assigns integers in a probabilistic sense to the quantitatively measured copy numbers, and is able to test simultaneously the association for all variants of interest in a regression framework. This integrative model can account for the uncertainty in copy number assignment and differentiate if the variation was *de novo* or inherited on the basis of posterior probabilities. For family studies, this model can accommodate the dependence within family members and among repeated CNV data. Moreover, the Mendelian rule can be assumed under this model and yet the genetic variation, including *de novo* and inherited variation, can still be included and quantified directly for each individual. Finally, simulation studies show that this model has high true positive and low false positive rates in the detection of *de novo* mutation.

## Introduction

Genetic variation in the human genome can take many forms. One is the abundance of submicroscopic copy number variations (CNVs) of DNA segments ranging from a kilobase to megabases (Iafrate et al., 2004; Sebat et al., 2004; Sharp et al., 2005; Tuzun et al., 2005). CNVs may exist as deletions, insertions, duplications, or complex multi-site variants (Redon et al., 2006). They may cause functional loss by means of dosage-related microdeletions, duplications, or altering regulatory regions of genes, and lead to phenotypic variation and modification of disease status (Stankiewicz and Lupski, 2002).

Two common biotechnologies, polymerase chain reaction (PCR) based and array based technique, have been available for CNV detection. In laboratories of PCR based detection (Bieche et al., 1998; Ponchel et al., 2003), the values of the threshold cycle (Ct) of the target and reference gene were first collected and then the difference ΔCt was used to infer the true copy number. Such technology is designed for targeted regions, and therefore is cost-saving and efficient, making it the method of choice for performing the repeated assessments for detecting targeted CNVs. The array-based techniques (Dhami et al., 2005; Sharp et al., 2005) can provide a genome-wide scan for novel CNVs. Such analysis was based on SNP intensity measures with log R ratios followed by clustering analysis for copy number assignment (Dellinger et al., 2010; Pinto et al., 2011). Although array-based technologies are useful in the discovery of large scale CNVs, the cost is relatively larger and hence may not be efficient in the validation of targeted CNVs. In addition to these two tools, the high throughput sequencing has recently become popular. However, the expense is even higher, as compared with the other two biotechnologies.

Because quantitative CNV values from PCR technology are derived based on signal intensity measures, ΔCt, and may be assessed repeatedly to control typing uncertainty, challenges arise in the exploration of CNV-disease associations. First, inference of discrete copy numbers is made based on continuous measurements repeatedly assessed. Using the nearest integer as the estimate may ignore systematic errors if they exist. Second, it is not straightforward to infer the copy number on each homologous chromosome when the only information collected is the sum of intensity measures on paired chromosomes. Information about copy numbers located in the same chromosome may contribute greatly in the investigation of Mendelian inconsistency and the evaluation of whether the variation is *de novo* or inherited. A further difficulty relates to the copy number assignment. Most existing analyses ignore these two types of uncertainty and treat the inferred integer as a constant value in later statistical tests of association. Such analyses may inflate the precision and induce false positives. Other challenges arise if the genetic markers are rare variants, leading to loss of statistical power.

To resolve these issues, a number of approaches have been suggested. Utilization of latent variables may help to evaluate the discrete values of CNVs, while incorporation of family information may reduce the uncertainty in CNV composition (Kosta et al., 2007; Wang et al., 2008). In addition, by simultaneously considering copy number determination and the test of association in an integrative way, both types of uncertainty mentioned above can be incorporated together and simultaneously addressed (Barnes et al., 2008). Furthermore, when dealing with rare variants, analysis should focus on multiple variants instead of a single one (Iyengar and Elston, 2007; International Schizophrenia, 2008; Stefansson et al., 2008; Walsh et al., 2008), such as by the pooling of rare variants with equal or unequal weights to achieve a larger power (Li and Leal, 2008; Madsen and Browning, 2009; King et al., 2010; Price et al., 2010).

The aim of this manuscript is to provide, via a Bayesian hierarchical model, a probabilistic evaluation of the strength of *de novo* mutation on rare CNVs as obtained by PCR methods and an association test in a schizophrenic family study. On the basis of the model proposed earlier (Kosta et al., 2007), this model incorporates mutation parameters, and can be tested for association in a regression framework. This model is able to accommodate the uncertainty in CNV determination and assignment, and the dependency among repeated CNV measurements per individual and within family members. All rare variants are considered as a set and the insertion and deletion can occur in offspring for evaluation of mutation. The design of the statistical model will be described in the following, with an illustration of a multiplex schizophrenia family study.

## Materials and methods

### Multiplex schizophrenia families and CNV

Schizophrenia, with a prevalence of 1% worldwide, is a complex disease with strong evidence of a genetic component. In Taiwan, the heritability has been estimated to be between 0.53 and 0.56 (Hwu et al., 2005; Tsai et al., 2010). Recently, CNVs have been shown to play an important role in Schizophrenia (Tam et al., 2009). For instance, CNVs in 1q21.1 were found in schizophrenic individuals with a frequency less than 1% (Stefansson et al., 2008), and a deletion form of CNV in 1q21.1 was observed to associate with an increased frequency in Japanese schizophrenia sufferers (Ikeda et al., 2010). These studies of CNVs in schizophrenic individuals conform to the assumption of “common disease rare variants” (Pritchard, 2001).

One key strategy to identify the associated rare variants is to select an “extreme” case group such as families with more than one affected relative (Bodmer and Bonilla, 2008; Stratton and Rahman, 2008). Hence we considered the study conducted by Hwu et al. (2005) who recruited schizophrenia patients and their first-degree relatives in the national Taiwan Schizophrenia Linkage Study (TSLS) in 1998–2002. All 2490 individuals from 607 families signed the informed consent forms. Among them, only 2462 subjects provided DNA specimens, including 1556 siblings (1242 affected, 79.8%) and 906 parents (65 affected, 7.2%). Their TSLS study was approved by both Internal Review Boards of Human Studies in the US Department of Health and Human Services and the National Taiwan University Hospital.

Three targeted CNV markers in two chromosomal regions, CNV1 and CNV2 on 2q22.1 (*HNMT* gene) and CNV3 on 1q21.1 (*GJA8* gene), were selected for genotyping by PCR-based technology for these schizophrenia sufferers and their families. Table 1 lists the information of the three CNV markers. The first region in 2q22.1 has been reported to show the most significant linkage with schizophrenia (Lien et al., 2011) and the second region in 1q21.1 was selected because its association with schizophrenia has been reported in other studies (Stefansson et al., 2008). The magnitudes of the Ct of the target and reference gene were recorded, respectively. The ΔCt was calculated as the difference between the Ct of the target gene and the Ct of the reference. This study adopted a two-stage qPCR procedure. In the first stage, two replicates were administered for each subject. Next, subjects and family members with an excessively high or low ΔCt value were selected for a second-stage genotyping consisting of an additional 4 replicates. The final predicted quantitative copy numbers (CN) were determined according to ΔCt with software CopyCaller v1.0, and the values were considered for later association analysis.

**Table 1:** **Information about the three CNV markers in TSLS**.

**CNV marker**	**Region (gene)**	**Assay ID**	**Probe sequence**
CNV1	2q22.1 (*HNMT*)	Hs01075733_cn	ATACATTATTGGACTTCCATTTGGA
CNV2	2q22.1 (*HNMT*)	Hs00435589_cn	CTCAACCATTCCACGGAACACCAGT
CNV3	1q21.1 (*GJA8*)	Hs02290971_cn	ATCCCTCCACTCCATTGCTGTCTCC

### Model for mutations in multiple CNVs

Let *y*_*ij*_ stand for the disease status of the *j*-th member in the *i*-th family with *y*_*ij*_ = 1 for affected subjects and *y*_*ij*_ = 0 for normal subjects. This *y*_*ij*_ is assumed to follow a Bernoulli distribution with parameter *p*_*ij*_, the probability of disease, which is linked to *C*_*ij*_, a function of copy number, and other explanatory variables *X*_*ij*_ through a logit function,

$$\text{logit}(p_{ij})=\alpha +\beta \times C_{ij}+\gamma \times X_{ij}+\beta_{i}.$$

The magnitude of the parameter β implies the strength of association between the probability of disease and the CNV of interest. Its inference will be based on the posterior distribution. Note that *C*_*ij*_ is a function of the actual copy number, *R*_*ij*_, not the copy number itself. The functional form can represent any biological interpretation that researchers aim to study. For instance, it can be an indicator function for “normal” alleles (when the CNV value is 2) at the single or multiple regions of interest,

$$C_{ij}=1-I_{\left\{2\right\}}(R_{ij})$$

where the integer *R*_*ij*_ is the true but unobserved copy number (the actual CNV value) of the *j*-th member in the *i*-th family. The value of the function *C*_*ij*_ becomes 1 if insertion or deletion appears (i.e., when *R*_*ij*_ is not 2); otherwise *C*_*ij*_ is 0 (i.e., *R*_*ij*_ is 2). When there are *L* multiple regions each with a copy number *R*_*lij*_ (*l* = 1, …, *L*), this function can be taken as

$$C_{ij}=1-\prod_{l=1}^{L}I_{\left\{2\right\}}(R_{lij})$$

for a pooling effect. The parameter γ is the regression coefficient of the covariate *X* and β_*i*_ stands for the family-specific random effect such that all subjects in the same family share a common baseline risk.

The true copy number, however, is not directly observable and has to be inferred from the quantitative CNVs. Let R^*^_ij_ (and R^*^_lij_) denote the quantitative CNV observation for the latent integer value *R*_*ij*_ (and *R*_*lij*_), where the index *l* is reserved for multiple CNVs and *l* is suppressed if only one single CNV is investigated. In the following, we illustrate with one CNV for simplicity of notation. Here R^*^_ij_ is assumed to follow a normal distribution with mean *R*_*ij*_ and variance σ^2^, R^*^_ij_ ~ *N*(*R*_*ij*_, σ^2^), where *R*_*ij*_ depends on paternal and maternal CNV values,

(1) $$\begin{array}{l} R_{ij}=k_{ij}^{f}\times \text{min}(a_{i1}^{f},a_{i2}^{f})+(1-k_{ij}^{f})\times \text{max}(a_{i1}^{f},a_{i2}^{f})\\ \;+k_{ij}^{m}\times \text{min}(a_{i1}^{m},a_{i2}^{m})+(1-k_{ij}^{m})\times \text{max}(a_{i1}^{m},a_{i2}^{m})\\ \;+I_{\left\{1\right\}}(\theta_{\text{insertion},ij})-I_{\left\{1\right\}}(\theta_{\text{deletion},ij}) \end{array}$$

For each family *i*, the *a*^*f*^_*ip*_, *p* = 1, 2 in (*a*^*f*^_*i*1_, *a*^*f*^_*i*2_) are the two CNV values of the father and (*a*^*m*^_*i*1_, *a*^*m*^_*i*2_) are those of the mother. These four values (*a*^*f*^_*i*1_, *a*^*f*^_*i*2_, *a*^*m*^_*i*1_, *a*^*m*^_*i*2_) are all non-negative integers. The *k*^*f*^_*ij*_ indicates whether the offspring inherits the smaller value of CNV from the father's CNV (*a*^*f*^_*i*1_, *a*^*f*^_*i*2_), while *k*^*m*^_*ij*_ indicates maternal inheritance (*a*^*m*^_*i*1_, *a*^*m*^_*i*2_). Both *k*^*f*^_*ij*_ and *k*^*m*^_*ij*_ follow a Bernoulli distribution with parameter 0.5. In addition, parents CNV values (*a*^*f*^_*i*1_, *a*^*f*^_*i*2_) and (*a*^*m*^_*i*1_, *a*^*m*^_*i*2_) are family-specific, and therefore the index *i* is necessary and the second subscript stands for two CNV values from paired chromosomes.

The last two indicator functions *I*_{1}_(θ_insertion, *ij*_) and *I*_{1}_ (θ_deletion, *ij*_) denote whether insertion or deletion occurs in the *j*-th member of the *i*-th family. The mutation parameter θ_insertion, *ij*_ (or θ_deletion, *ij*_) for this individual is 1 if the locus is a copy gain (or copy loss). The inclusion of these two parameters can resolve Mendelian inconsistency between the CNV values of parents and of offspring when mutation occurs. These two parameters are assumed to follow a Bernoulli distribution with a parameter of small value.

The quantitative CNV observations R^*^_ij_ for parents are also normally distributed R^*^_ij_ ~ *N*(*R*_*ij*_, σ^2^), but the mean parameter *R*_*ij*_ is now *R*_*ij*_ = *a*^*f*^_*i*1_ + *a*^*f*^_*i*2_ for father and *R*_*ij*_ = *a*^*m*^_*i*1_ + *a*^*m*^_*i*2_ for mother where no mutation is allowed in parents. These *a*^*f*^_*i*1_, *a*^*f*^_*i*2_, *a*^*m*^_*i*2_, *a*^*m*^_*i*2_ all follow a Poisson distribution.

When replicates were collected, the CNV observations R^*^_ij_ will be written as R^*^_ij,k_ where *k* = 1, …, *n*_*ij*_ with *n*_*ij*_ indicating the number of replications for this individual. For this TSLS study, *i* = 1,…, 607 for the 607 families, *j* = 1, …, *n*_*i*_ for the size of each family and *n*_*ij*_ is either 2 or 6.

The statistical inference is based on posterior samples from Markov chain Monte Carlo methods (MCMC) obtained with OpenBUGS (Lunn et al., 2009). The code is detailed in the supporting information. The chain contains 50,000 iterations following a burn-in of 50,000 samples to reduce the impact from initial values and the final posterior samples were derived at a thinning rate of 10 to reduce the dependence among iterations.

## Results

Among the 607 families, 598 (98.5%) families have at least one parent's information available, and 605 families have at least two children. The distribution is shown in Table 2. In the first stage, every individual was genotyped twice for these CNV regions. Any individual whose ΔCt was beyond mean ± 3 *SD* was genotyped again, along with his/her family members. A total of 31 subjects from 8 families, 20 subjects from 5 families, and 62 subjects from 15 families were selected for the second-stage genotyping for the CNV1 and CNV2 in the *HNMT* gene on 2q22.1 and CNV3 in the *GJA8* gene on 1q21.1, respectively.

**Table 2:** **Numbers of families with different numbers of children and parents recruited in TSLS (607 families)**.

**No. of parents**	**No. of children**				
	**1**	**2**	**3**	**≥4**	**Sum**
0	1	5	3	0	9
1	0	11	262	18	291
2	1	277	24	5	307
Total	2	293	289	23	607

**Figure 1:**
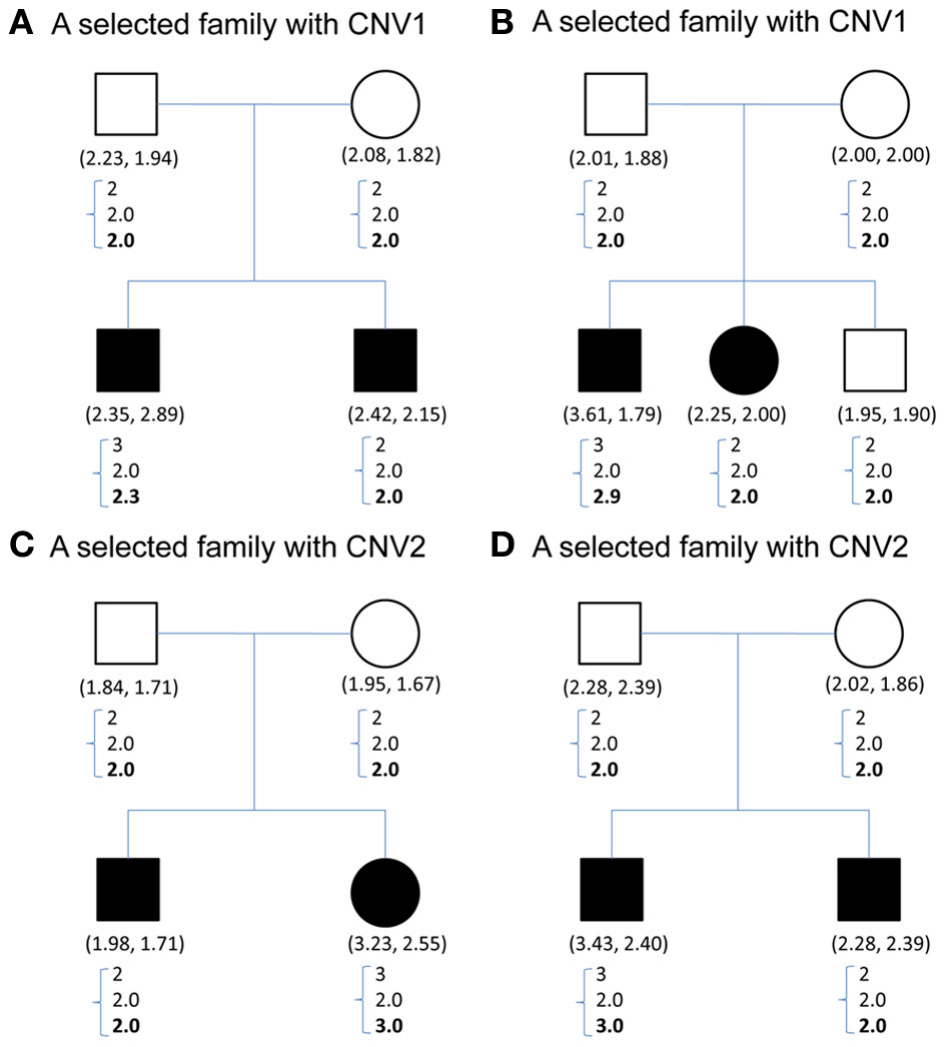
**(A–D)** Contain CNV information for four sample families selected from TSLS. Solid symbols indicate schizophrenia sufferers. Below each symbol, the two numbers in parentheses show the replicate CNV measures. Below the parentheses, there is a column of three numbers. The first one represents the predicted value, the closest integer to the average of the replicate CNV measures; the second represents the posterior mean of the latent copy number under the model without mutation parameters; and the third number in boldface represents the posterior mean of the latent copy number under the model with mutation parameters. **(A,B)** Contain the CNV1 information, and **(C,D)** are for CNV2.

Figure 1 demonstrates the variable copy number assignment within a pedigree of four selected families in TSLS. In Figure 1A, the copy numbers of parents were both assigned 2, the nearest integer to the average of the quantitative CNV values. However, the copy numbers of their two children were 3 and 2, respectively, leading to Mendelian inconsistency. With the model introduced in the Methods section but containing no parameter for copy gain θ_insertion, *ij*_ or for copy loss θ_deletion, *ij*_, the assigned copy number was 2 for the parents and two children. Although this result satisfies the Mendelian rule, it does not allow the possibility of genetic variation or mutation to occur in the first child, whose copy number may be 3 instead of 2.

**Figure 2:**
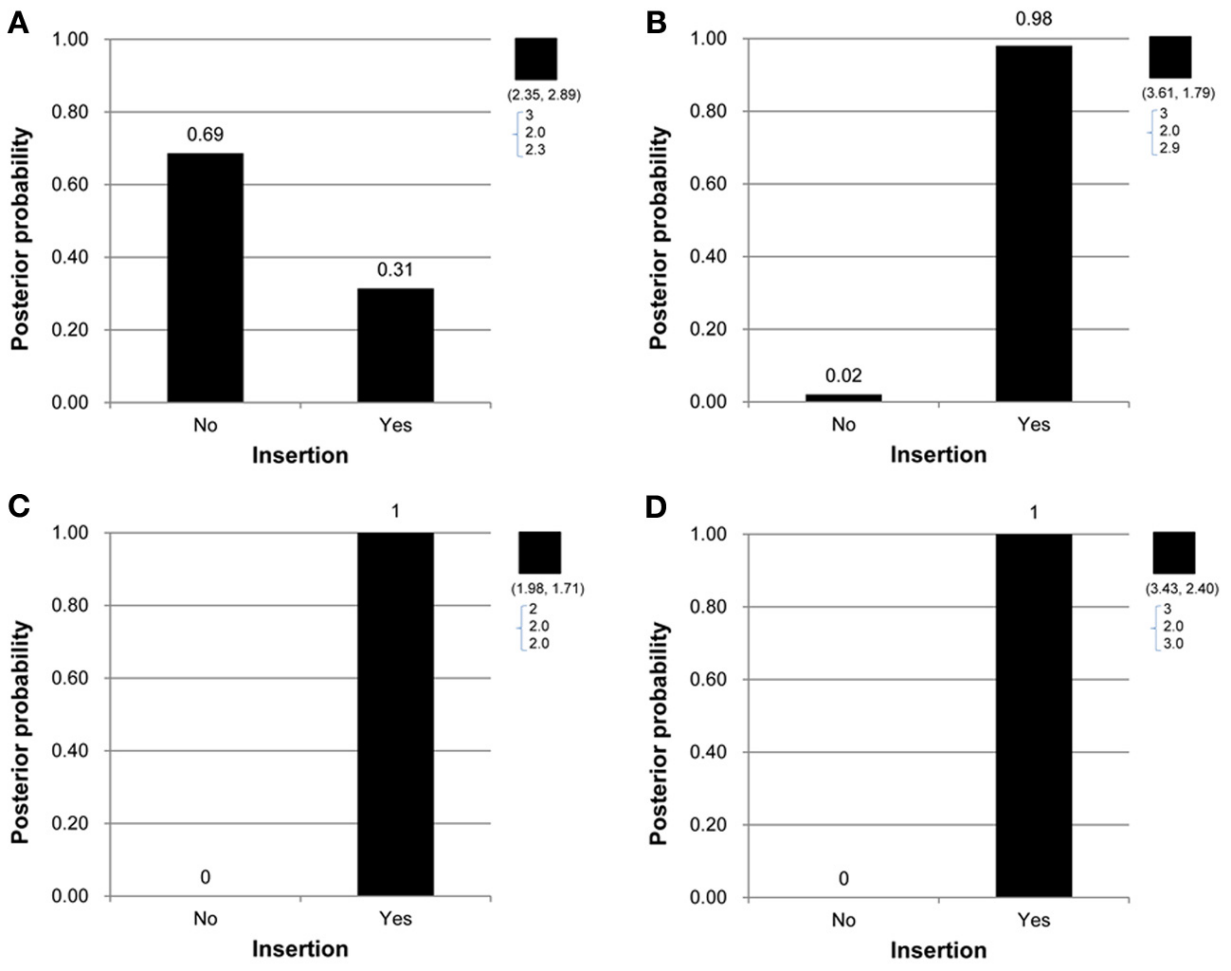
**(A–D)** Are the discrete posterior probabilities of the binary insertion parameter for four selected offspring in four families in Figures 1A–D.

Under the proposed model containing the mutation parameters, the posterior mean obtained for the latent copy number of the first child in Figure 1A was 2.3 and the mode was 2. In other words, the posterior probability of having a copy gain mutation for this child was 30%, which is not a small number and hence implies mutation with some degree of evidence. Similarly, the first child in Figure 1B, the second child in Figure 1C, and the first child in Figure 1D all provided evidence of mutation. Out of the 307 families (restricted to families where both parents' information was available), this model detected in these four families that a copy gain variation has occurred in either CNV1 or CNV2. Figure 2 demonstrates the discrete posterior distributions of the latent copy numbers for these four individuals, respectively.

**Figure 3:**
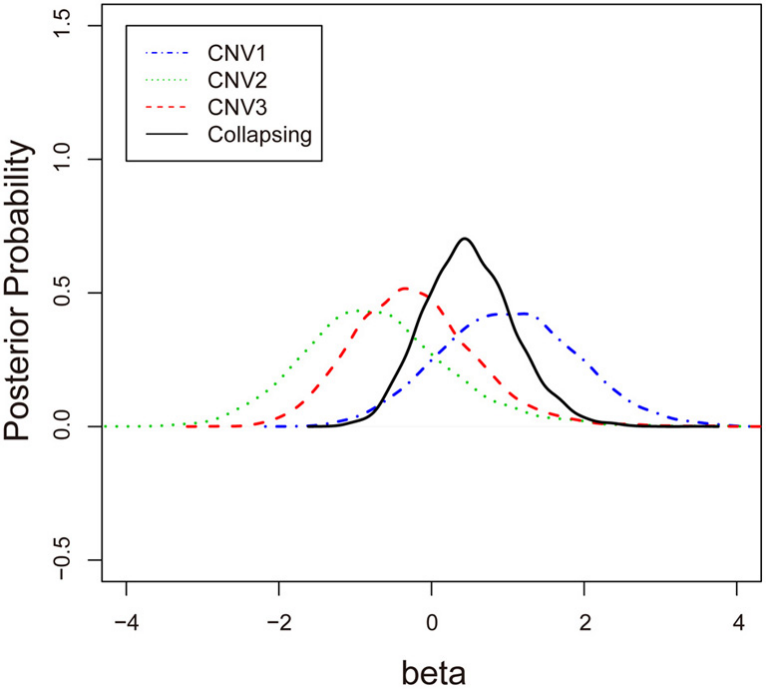
**The posterior probabilities of β under three single-CNV models and one multiple-CNV model**.

To examine the association between the individual CNV or the collapsing variants and schizophrenia, Table 3 lists the posterior means and standard deviations of the genetic effect under the single- and multiple-marker model, respectively. Subjects with CNV1 variant were more likely to have schizophrenia. The posterior probability of such risk was as high as 86.9%. In contrast, subjects with CNV2 or CNV3 variants were less likely to have schizophrenia, with posterior probabilities of 20.8 and 35.9%, respectively. When the three CNV3 were collapsed, the pooling effect on schizophrenia was large, with odds ratio exp(0.49) = 1.63, and a posterior probability of 79.7%. Figure 3 demonstrates the densities of β, i.e., the strength of association, under both the single CNV and the multiple CNV model. Clearly, the model with CNV1 and the one with collapsing variants show a larger degree of association.

**Table 3:** **The posterior mean and standard deviation of β, and the posterior probability of (β > 0) under the three single CNV marker models and the model with collapsing CNVs**.

	**Mean (se)**	***P* (β > **0**) (%)**
CNV1	1.02 (0.90)	86.9
CNV2	−0.72 (0.98)	20.8
CNV3	−0.23 (0.82)	35.9
Collapsing 3 CNVs	0.49 (0.59)	79.7

### Simulation studies

We conducted simulation studies to evaluate the performance of this Bayesian hierarchical model in detecting *de novo* mutation. The CNV markers of the parents were first generated, with the frequency of insertion in this marker fixed at 0.01. Next, the copy number of this CNV marker for the offspring was generated according to the basic Mendelian rule, and the *de novo* mutations were assigned a 0.005 probability of copy gain and a 0.005 probability of copy loss. The observed quantitative CNV values were determined by the generated copy number plus an error term from a normal distribution with zero mean and a fixed standard deviation. The number of families was fixed at 200, where the number of family members in each family was fixed at 4. In the different simulation settings, the standard deviation was fixed at 0.15, 0.2, or 0.25. Under each setting the number of replications was 1000.

**Figure 4:**
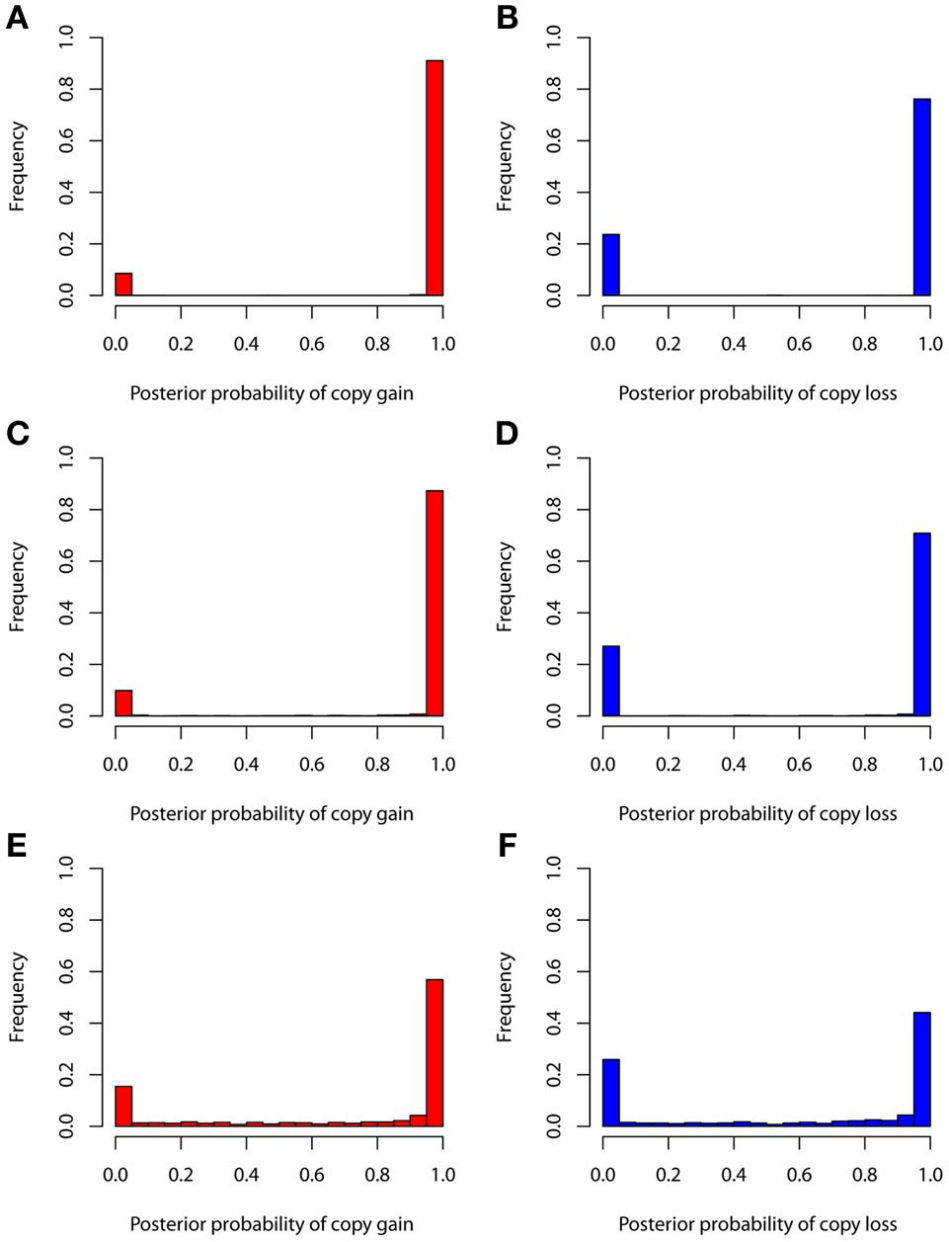
**True positive rates of detecting copy gain and copy loss under various standard deviations. (A)** For copy gain when *SD* = 0.15, **(B)** for copy loss when *SD* = 0.15, **(C)** for copy gain when *SD* = 0.20, **(D)** for copy loss when *SD* = 0.20, **(E)** for copy gain when *SD* = 0.25, and **(F)** for copy loss when *SD* = 0.25.

**Figure 5:**
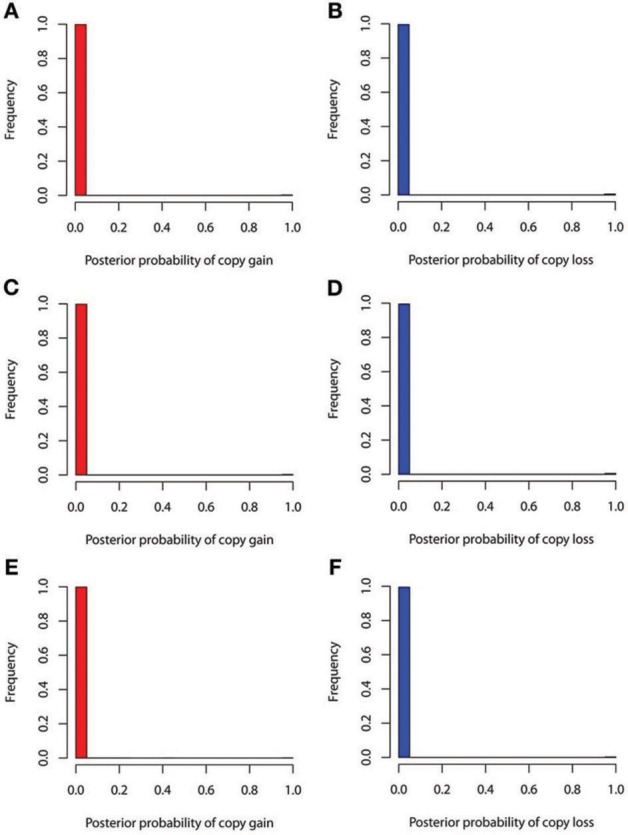
**False positive rates of detecting copy gain and copy loss under various standard deviations. (A)** For copy gain when *SD* = 0.15, **(B)** for copy loss when *SD* = 0.15, **(C)** for copy gain when *SD* = 0.20, **(D)** for copy loss when *SD* = 0.20, **(E)** for copy gain when *SD* = 0.25, and **(F)** for copy loss when *SD* = 0.25.

**Figure 6:**
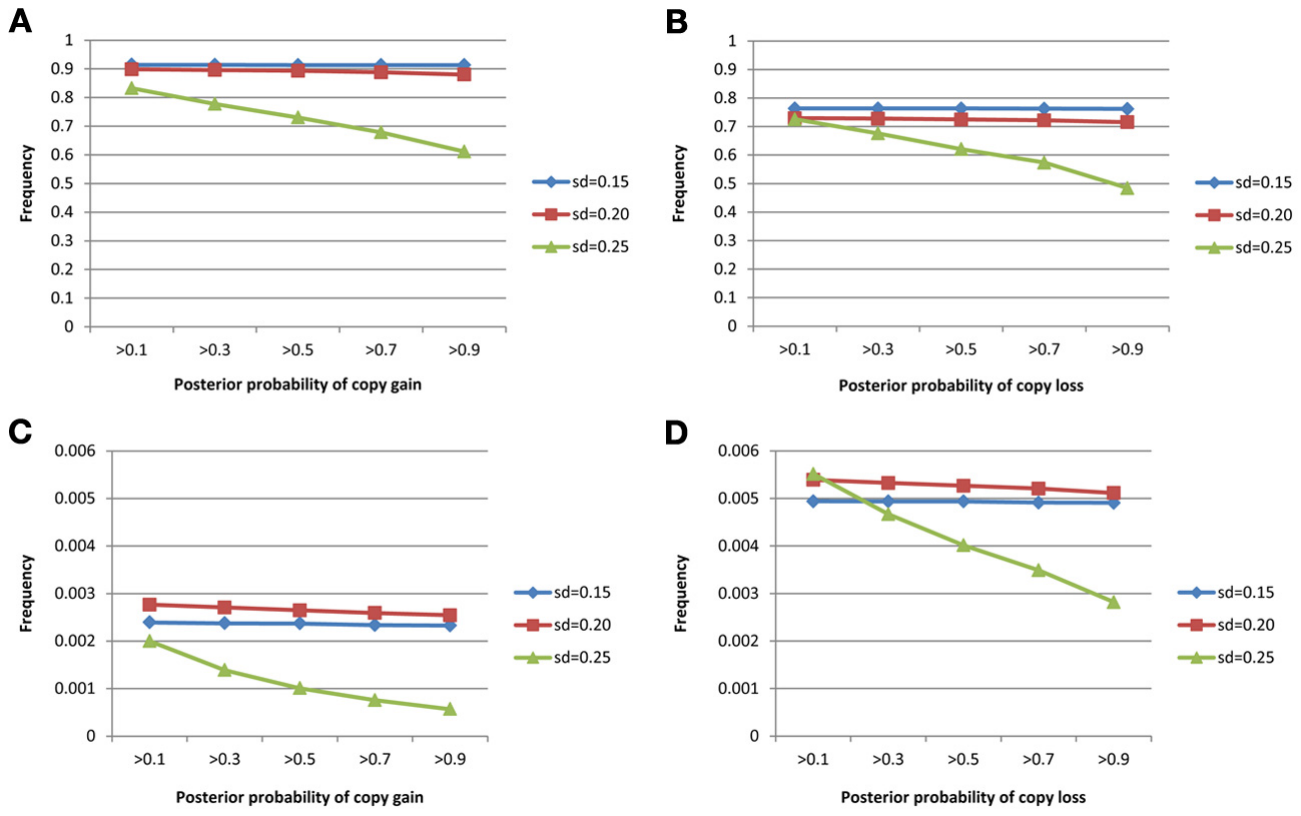
**True positive rates of detecting copy gain/loss and false positive rates of detecting copy gain/loss with different cut-points for determination of the *de novo* mutation. (A)** For true positive of copy gain, **(B)** for true positive of copy loss, **(C)** for false positive of copy gain, and **(D)** for false positive of copy loss.

The true positive rates of detecting copy gain and copy loss under various values of standard deviations are shown in Figure 4. It is apparent that the posterior probability of correctly detecting the mutation was very large regardless of the standard deviation. For those offspring without *de novo* CNV, the posterior probability of no detection was largely concentrated at 0 (Figure 5). Under different cut-off values for correct detections, the frequencies of true positive and false positive detection are shown in Figure 6. For a standard deviation set at 0.15 and 0.2, the true positive rate of detection for copy gain is about 0.9 and the true positive rate of detection for copy loss is about 0.7–0.8 regardless of the cut-off points. For a standard deviation set at 0.25, the threshold values need to be small, otherwise such large laboratory error will dominate the precision of the current method.

**Figure 7:**
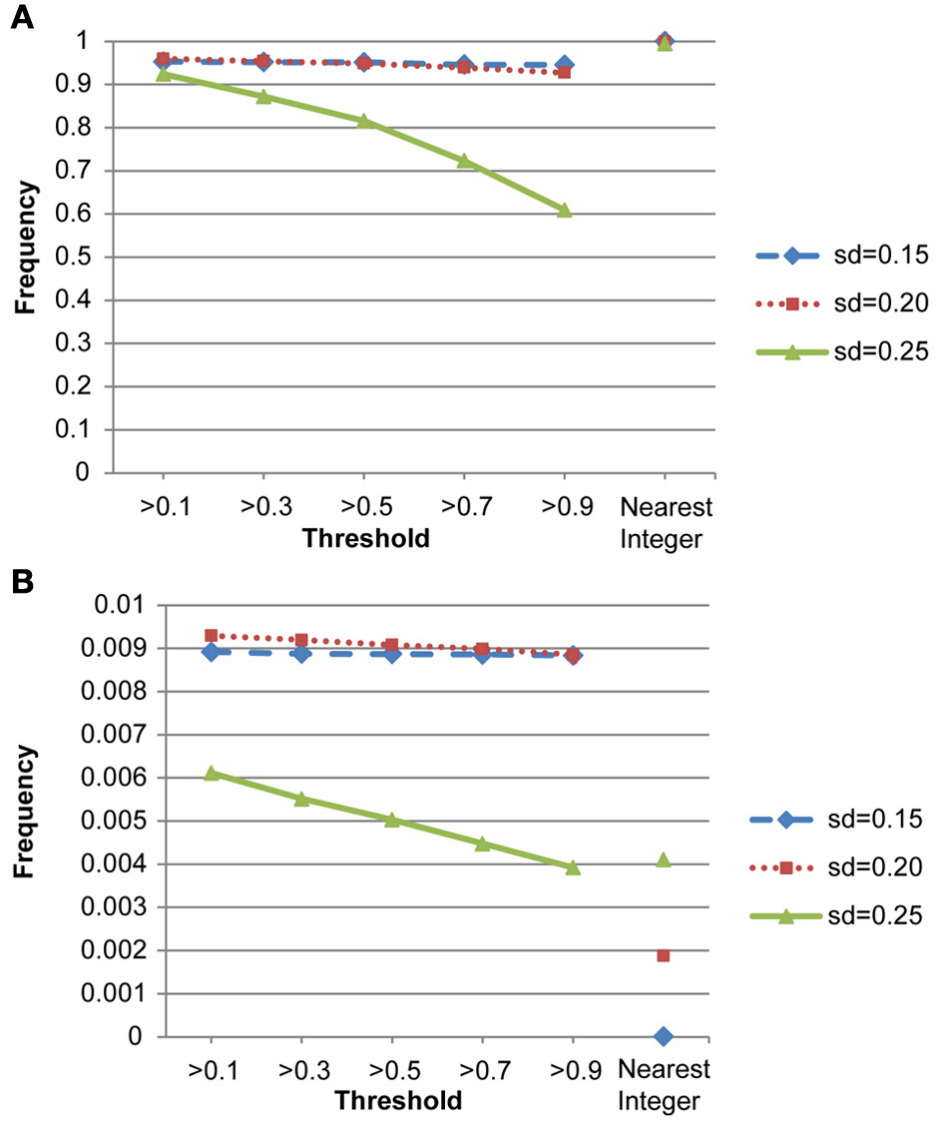
**(A)** True positive rates of detecting *de novo* mutation families by the proposed model and the nearest integer method (the three points in the rightmost column). **(B)** False positive rates of detecting *de novo* mutation families by the proposed model and the nearest integer method (the three points in the rightmost column).

We also conducted a simulation study to compare the performance of the nearest integer method in identifying families with *de novo* mutations. In other words, if the focus is no longer in the individual who may carry the mutations but in the family which may contain mutations, then this may be indicated by the failure to satisfy the Mendelian rule for the CNV estimates within family members. In this case, we applied the nearest integer method first to estimate the true CNVs, and then examine if these estimates satisfy the Mendelian rule. If not, then a mutation may occur in at least a member of this family. However, the information of the type of mutations, gain or loss, as well as the probability of mutations will not be available under the non-parametric nearest integer method. For the proposed Bayesian model, as long as one member's posterior probability of θ_insertion, *ij*_ or θ_deletion, *ij*_ exceeds the threshold value (0.1, 0.3, 0.5, 0.7, or 0.9), then this family is counted as a family with mutations. Table 4 and Figure 7 list the true positive rates and false positive rates of detecting *de novo* mutation families under various values of standard deviations. It is obvious that the nearest integer method has the largest true positive rates (larger than 0.99). The proposed Bayesian model performed better when the standard deviation was set at 0.15 or 0.20, with a true positive rate larger than 0.93, and was not robust for greater genotyping variation. For false positive rates, both methods had values smaller than 0.01 but the nearest integer method clearly outperformed with values less than 0.005. Although the nearest integer method can identify correctly which family contains the *de novo* mutation, it cannot estimate the composition of CNVs in the paired chromosomes, and hence cannot evaluate which member carried the mutation, or the type of mutations.

**Table 4:** **True positive rate and false positive rate of detecting mutation family under various standard deviation of PCR-based typing by the Bayesian hierarchical model and the method of nearest integer**.

	**Threshold**					**Nearest**
	**>0.1**	**>0.3**	**>0.5**	**>0.7**	**>0.9**	**integer**
**TRUE POSITIVE RATE**						
*SD* = 0.15	0.9528	0.9518	0.9513	0.9458	0.9453	1.0000
*SD* = 0.20	0.9593	0.9538	0.9485	0.9390	0.9273	0.9985
*SD* = 0.25	0.9238	0.8718	0.8155	0.7228	0.6090	0.9940
**FALSE POSITIVE RATE**						
*SD* = 0.15	0.0089	0.0089	0.0089	0.0089	0.0088	0.0000
*SD* = 0.20	0.0093	0.0092	0.0091	0.0090	0.0089	0.0019
*SD* = 0.25	0.0061	0.0055	0.0050	0.0045	0.0039	0.0041

## Discussion

This Bayesian hierarchical model is designed to simultaneously detect the *de novo* CNV by PCR-based technology and to test for its association with the disease of interest. This integrative model can account for the uncertainty in copy number assignment and quantify the strength of the evidence that the variation was *de novo* or inherited, on the basis of posterior probabilities, by allowing the insertion and deletion to occur in offspring. To test the association between CNVs and disease status, Kosta's Bayesian model (Kosta et al., 2007) analyzed transmissions of variational copy numbers in affected and non-affected siblings, without the use of parents' disease information. The Bayesian model proposed here can take into account the disease information of both parents and siblings. For the TSLS study, since schizophrenia is a complex disease with a diverse spectrum of severity, the recruited parents may be relatively healthy as compared to those not recruited. Therefore, to avoid ascertainment bias, here we used CNV data from both parents and children to assess chromosome inheritance, but used only the disease status of children in the association test. When ascertainment is not an issue, all information should be included in the model for analysis.

In addition to the inference of true copy number assignment, this Bayesian hierarchical model incorporates the possibility that insertion and deletion might occur in offspring, so that variation can be differentiated as *de novo* or inherited. Our simulation studies show that this Bayesian model performed with high true positive and low false positive rates in detection of *de novo* mutation. In addition, false positives in detecting *de novo* mutation with this model were low.

When applying this model, one should be careful about the prior specification of the mutation rate. The rule of thumb is to specify the mutation rate following a Bernoulli distribution with a parameter not larger than the reciprocal of sample size. This allows for the possibility of *de novo* CNV occurring and reduces the chances of a false positive. In addition, it makes possible the assumption of different effects for *de novo* CNV and inherited CNV in this model, when more prior knowledge is available.

Some limitations of this article are noted here. First, as discussed earlier, this proposed model cannot consider the case when ascertainment occurs. In other words, when the collection of information depends on how the subjects were ascertained, such as an early-onset disease, then this model cannot be applied directly. Second, if the research interests lie simply in the CNV assignment (the *R*_*ij*_ in the model) and families carrying mutations, and not in the composition of CNVs (i.e., the components in the right hand side of Equation (1) in each of the paired chromosomes, then the existing method of nearest integer already works well. As illustrated in the last simulation study, the CNV estimates from the nearest integer method are accurate and thus a logistic regression without inference on mutation can be applied based on these CNV estimates. In this case, there would be no need to employ the proposed Bayesian model. Third, any mutation identified based on this statistical model requires further validation in laboratory research. The results here do not imply causality but provide possible targets of association. Fourth, since no other research considered the probability of mutation in analysis, we did not compare this proposed approach with other existing methods in the simulation studies. The comparison we conducted, however, is with the method of nearest integer to evaluate the CNV assignment and identify families with mutation events. To the best of our knowledge, the proposed approach is the first model that simultaneously considers the inference of copy number assignment, composition of copy numbers inherited from parents, and inclusion of probability of mutation in each offspring. More studies are needed in this research topic.

This Bayesian model assumes that all rare CNVs share effects in the same direction, and thus the collapsing approach can be carried out for the association test. In cases where some of the rare CNVs are risk factors while others are protective, such a pooling approach needs modification. Further research would be worth pursuing.

### Conflict of interest statement

The authors declare that the research was conducted in the absence of any commercial or financial relationships that could be construed as a potential conflict of interest.

## Appendix

**Supporting Information: OpenBUGS code for the Bayesian model**

model

{

for (i in 1:N){   #N: number of families

       beta.I[i]~dnorm(0.0,tau.I)     # family-specific random effect

for (j in 1:2){      # specification for father and mother's risk for each family

       CN1[i,j] <- no11[i,j]+no12[i,j]       # CNVs from paired chromosomes

no11[i,j] ~ dpois(1)

no12[i,j] ~ dpois(1)

              disease[i,j] ~ dbern(p[i,j])

               logit(p[i,j])<-alpha+beta*CNl[i,j]+beta.I[i]

for (k in 1:2) {      # two repeated measurements for quantitative CNVs

           CNVall[i,j,k] ~ dnorm (CN1[i,j], tau.CN)            # quantitative CNV

       }

   }

for (j in 3:4){     #2 specification for offspring's risk

  CN1[i,j]<-

  equals(tau11[i,j],0)*min(no11[i,1],no12[i,1])

  +equals(tau11[i,j],1)*max(no11[i,1],no12[i,1])

  +equals(tau12[i,j],0)*min(no11[i,2],no12[i,2])

  +equals(tau12[i,j],1)*max(no11[i,2],no12[i,2])

  +equals(plus1[i,j],1)*1-equals(minus1[i,j],1)*1

       tau11[i,j] ~ dbern(0.5)      # indicator for inherited CNV from father

       tau12[i,j] ~ dbern(0.5)      # indicator for inherited CNV from mother

       plus1[i,j]~ dbern      # insertion parameter, distribution needs to be specified

       minus1[i,j]~ dbern      # deletion parameter, distribution needs to be specified

              disease[i,j] ~ dbern(p[i,j])

              logit(p[i,j])<-alpha+beta*CNl[i,j]+beta.I[i]

for (k in 1:2) {

       CNVall[i,j,k] ~ dnorm (CN1[i,j], tau.CN)

       }

  }

}

alpha~ dnorm         # regressions coefficient, distribution needs to be specified

beta~ dnorm         # regression coefficient, distribution needs to be specified

tau.CN~ dgamma         # precision in distribution of quantitative CNV, distribution needs to be specified

var.CN<-1/tau.CN

tau.I~ dgamma         # precision in family random effect, distribution needs to be specified

var.I<-1/tau.I }
